# Use of unscheduled care in the last year of life for people with multiple long-term health conditions: a retrospective cohort study of 299 361 decedents

**DOI:** 10.3399/BJGPO.2025.0049

**Published:** 2026-02-25

**Authors:** Sarah P Bowers, Maureen Ward, Margaret C Weir, Sarah EE Mills, Linda Williams, Joanna Bowden, Frances Quirk

**Affiliations:** 1 School of Medicine, University of St Andrews, St Andrews, UK; 2 Fife Community Advisory Council, School of Medicine, University of St Andrews, St Andrews, UK; 3 Edinburgh Clinical Trials Unit, Usher Institute, University of Edinburgh, Edinburgh, UK; 4 NHS Fife, Kirkcaldy, UK

**Keywords:** multimorbidity, palliative care, primary health care, community care, emergency services, after-hours care

## Abstract

**Background:**

People living with and dying from multiple long-term conditions (MLTCs) are high users of healthcare services. Unscheduled care, the unplanned use of healthcare services, rises dramatically in the last year of life, likely reflecting unmet needs.

**Aim:**

To characterise Scotland-based decedents with MLTCs in their last year of life and explore the relationship between characteristics and unscheduled care usage over that year.

**Design & setting:**

Retrospective cohort study of all individuals who died in Scotland between 1 January 2017 and 31 December 2021.

**Method:**

Data were linked across routine NHS Scotland datasets. Associations between sociodemographic factors, MLTCs, and unscheduled care usage in the last year of life were explored through binary logistic regression.

**Results:**

In total, 299 361 individuals died in Scotland between 1 January 2017 and 31 December 2021, of whom 136 593 (45.6%) had ≥2 long-term health conditions leading into their last year of life. More decedents with MLTCs (97.1%) used unscheduled care compared with those without (95.6%). When adjusted for sociodemographic factors, those with MLTCs were more likely to use unscheduled care (adjusted odds ratio 1.51, 95% confidence interval = 1.45 to 1.57).

**Conclusion:**

People dying with MLTCs had particularly high use of unscheduled care in the last year of life, likely reflecting unmet need. Anticipating and addressing these needs, through usual care providers, could reduce avoidable use of unscheduled care.

## How this fits in

Previous research has looked at unscheduled care usage for people living with single conditions. This study highlights that people with multiple long-term conditions (MLTCs) use more unscheduled care in the last year of life, and differently, compared with those with fewer conditions. Further research should explore why age and socioeconomic factors are associated with particular patterns of unscheduled care use. Inadequate availability and accessibility of planned and urgent in-hours care and support are potential drivers of unscheduled care usage. Further research should also explore difficulties that individuals have accessing care and the extent to which more proactive support for people in the last year of life, particularly those with MLTCs, could reduce their unscheduled care use.

## Introduction

People living with and dying from MLTCs (the coexistence of ≥2 long-term conditions)^1^ are high users of healthcare services, including so-called ‘unscheduled care services’, which refers to the unplanned use of healthcare services.^2^ Unscheduled care usage may reflect unmet care needs, which have not been foreseen by patients’ existing care providers.^3^ Often, unscheduled care usage is driven by a lack of coordinated care or an inability of other services to meet that person’s urgent needs.^4^ Uncoordinated care is experienced by people with MLTCs throughout their life and there are concerns that our healthcare system, a predominantly single-condition model, fails to reflect and respond to their multifaceted needs.^5^

Unscheduled care usage rises dramatically towards the end of life, with much of this delivered in the out-of-hours period.^6–8^ The associated costs also rise exponentially, particularly in the final month of life, for healthcare services, patients, and caregivers.^9,10^ Recent estimates show that people in the last year of life account for nearly 10% of all hospital costs, with additionally high expenditure on social care and welfare.^11^ However, the provision of care entails costs and burdens beyond the monetary, and highly medicalised pathways often conflict with people’s priorities at the end of life, which, for many, include minimising time spent in hospital and only receiving interventions that could realistically improve their quality of life.^12^ This aligns with principles of Realistic Medicine, a value-based healthcare approach — where value is considered in its multifaceted meaning — ensuring interventions delivered align with patients’ personal priorities and values.^13^

Most studies of unscheduled care use towards the end of life characterise individuals according to conditions recorded on their death certificates, reflecting cause(s) of death, and not necessarily the conditions that they were living with over preceding months and years.^6,7^ Although it has been predicted that more people will be living with and dying from MLTCs over time,^14^ existing studies have not examined the population-level distribution of MLTCs within a dying population, nor the impact of these on unscheduled care usage. The aim of this study was to characterise a population of Scotland-based decedents with MLTCs who were in their last year of life and to describe their unscheduled care usage over that year, including the frequency, type, and timing of unscheduled care use.

## Method

### Design

A retrospective cohort study was conducted using routinely collected health data describing patterns of unscheduled care usage in the last year of life, with a focus on people living with MLTCs. Results are reported in accordance with the REporting of studies Conducted using Observational Routinely-collected health Data checklist.^15^

### Setting

Health care within Scotland is underpinned by the NHS, a publicly funded, free at the point of access system. Patients within Scotland access health care via scheduled appointments, through GPs in primary care or outpatient appointments in secondary care, or via unscheduled care if more urgent matters arise. Unscheduled care in NHS Scotland comprises:

- general practice out of hours (GPOOH), which refers to primary care services available when usual primary care teams are unavailable;
- NHS 24, which is a national telephone advice line, for non-urgent conditions;
- Scottish Ambulance Service (SAS), which is an ambulance service available via direct contact for assessment and management of urgent conditions and/or conveyance to hospital;
- accident and emergency (A&E), where emergency departments assess and manage urgent conditions; and
- hospital admissions that are arranged by primary or secondary care providers, or other unscheduled care services.

### Population

All decedents aged ≥18 years who died in Scotland between 1 January 2017 and 31 December 2021 were included. Unscheduled care usage was examined for 12 months preceding death for each decedent.

### Data sources

A range of datasets were included: death certification records; hospitalisations (acute hospital and mental health specialties); cancer registrations; and the Scottish Unscheduled Care Datamart (GPOOH, NHS 24, SAS, and A&E attendances). Data were linked via the Community Health Index, a unique patient identifier used throughout NHS Scotland. All data were accessed and analysed in the National Safe Haven, a trusted research environment.

### Variables: demographics

Demographic data were captured from contacts with health services within the datasets for 5 years before death. These data were obtained cumulatively to maximise data completeness. These included sex, ethnicity, deprivation using the Scottish Index of Multiple Deprivation (SIMD) 2020^16^ (five-level area-based socioeconomic deprivation classification), and rurality using the Scottish Government Urban Rural Classification^17^ (eight-level rurality classification). Age at death and place of death were obtained from death records.

### Variables: MLTCs

MLTCs was defined as the co-existence of ≥2 long-term conditions before the last year of life and identified through International Classification of Diseases, 10th Revision (ICD-10) codes of conditions obtained cumulatively;^1,18^ including conditions diagnosed at least 12 months before death ensured these conditions were chronic.^18^ Included conditions were selected primarily through published consensus-derived lists,^18–20^ with adaptations made because of variable availability within datasets, using the clinical expertise of the author group (see Supplementary Table S1).

### Outcome

The primary outcome of interest was any use of unscheduled care in the last year of life as a binary outcome. Additionally, type of unscheduled care used was categorised, including identifying contacts in the out-of-hours period, according to time of first contact with that service, outside of Monday–Friday 9 am–5 pm and UK public holidays (admissions data were limited to only look at day of attendance).

### Statistical analysis

Medians and interquartile ranges (IQRs) were calculated for usage of each unscheduled care service owing to non-normality of the data distribution. Rates per 1000 deaths for cohorts with and without MLTCs are also presented. Binary logistic regression was used to identify associations between use of unscheduled care services, demographic factors, and presence of MLTCs, and presented as adjusted odds ratios (aORs) with 95% confidence intervals (CIs). Missing data were excluded from regression models. Data were analysed using R (version 4.4.2), with finalfit used for logistic regression.

### Patient and public involvement

The project Public Advisory Group (PAG) comprised public members with lived experience of caring for family members in their last year of life. The PAG was integral to this study, shaping research questions, co-authoring data applications, interpreting findings, and co-authoring this publication. Their involvement ensured that lived experience has been represented and findings are meaningful to the wider population.

## Results

### Demographics of cohort

In total, 299 361 individuals aged ≥18 years died in Scotland between 1 January 2017 and 31 December 2021 (Table 1). A high proportion of decedents were aged ≥80 years (53.0%) and 50.7% were female. Around one-quarter of decedents (24.4%) were in the most deprived socioeconomic quintile (SIMD 1). Most decedents lived in highly urban areas (35.4% in UR8-1 and 35.7% in UR8-2). Only 0.8% of decedents were of non-White ethnicity, although ethnicity data were unavailable for 14.4%.

**Table 1.** Demographics of decedent cohort

Characteristic	<2 long-term conditions, *n* (%), *N =* 162 768	≥2 long-term conditions, *n* (%), *N =* 136 593	Total decedent population, *n* (%), *N =* 299 361
**Sex**			
Female	80 921 (49.7)	70 830 (51.9)	151 751 (50.7)
Male	81 847 (50.3)	65 763 (48.1)	147 610 (49.3)
**Age, years**			
18–29	1765 (1.1)	320 (0.2)	2085 (0.7)
30–39	3280 (2.0)	1027 (0.8)	4307 (1.4)
40–49	6261 (3.8)	2664 (2.0)	8925 (3.0)
50–59	13 002 (8.0)	6416 (4.7)	19 418 (6.5)
60–69	22 721 (14.0)	14 047 (10.3)	36 768 (12.3)
70–79	37 844 (23.3)	31 426 (23.0)	69 270 (23.1)
≥80	77 895 (47.9)	80 693 (59.1)	158 588 (53.0)
**SIMD 2020 quintile**			
1 (most deprived)	38 507 (23.7)	34 546 (25.3)	73 053 (24.4)
2	35 805 (22.0)	31 448 (23.0)	67 253 (22.5)
3	33 736 (20.7)	28 212 (20.7)	61 948 (20.7)
4	29 014 (17.8)	23 063 (16.9)	52 077 (17.4)
5 (least deprived)	25 380 (15.6)	19 259 (14.1)	44 639 (14.9)
Not known	326 (0.2)	65 (0.05)	391 (0.1)
**Urban Rural Classification 2020^a^**			
UR8-1 (most urban)	56 904 (35.0)	48 979 (35.9)	105 883 (35.4)
UR8-2	57 415 (35.3)	49 492 (36.2)	106 907 (35.7)
UR8-3	14 326 (8.8)	11 850 (8.7)	26 176 (8.7)
UR8-4	3122 (1.9)	2689 (2.0)	5811 (1.9)
UR8-5	2514 (1.5)	2096 (1.5)	4610 (1.5)
UR8-6	18 295 (11.2)	13 973 (10.2)	32 268 (10.8)
UR8-7	4926 (3.0)	3766 (2.8)	8692 (2.9)
UR8-8 (most rural)	4940 (3.0)	3683 (2.7)	8623 (2.9)
Not known	326 (0.2)	65 (0.05)	391 (0.1)
**Ethnicity**			
African, Black, or Caribbean	147 (0.1)	92 (0.1)	239 (0.1)
Asian	765 (0.5)	791 (0.6)	1556 (0.5)
Mixed, multiple, or other ethnic groups	323 (0.2)	195 (0.1)	518 (0.2)
White	127 473 (78.3)	126 470 (92.6)	253 943 (84.8)
Not known	34 060 (20.9)	9045 (6.6)	43 105 (14.4)
**Place of death^b^**			
Home	54 001 (33.2)	34 184 (25.0)	88 185 (29.5)
Hospital	78 337 (48.1)	68 055 (49.8)	146 392 (48.9)
Nursing or care home	30 351 (18.6)	34 320 (25.1)	64 671 (21.6)
Other	78 (0.05)	34 (0.02)	112 (0.04)

^a^Urban Rural Classification categories: 1 = ≥125 000 people; 2 = 10 000–124 999 people; 3 = 3000–9999, within a 30-minute drive to a settlement of ≥10 000; 4 = 3000–9999, with a 30–60-minute drive to a settlement of ≥10 000; 5 = 3000–9999, with >60-minute drive to a settlement of ≥10 000; 6 = <3000 people, within a 30-minute drive to a settlement of ≥10 000; 7 = <3000 people, with a 30–60-minute drive to a settlement of ≥10 000; and 8 = <3000 people, with >60-minute drive to a settlement of ≥10 000. ^b^Data missing for 1 decedent. SIMD = Scottish Index of Multiple Deprivation.

### MLTCs within the cohort

Decedents had between 0 and 14 recorded conditions, with 136 593 decedents (45.6%) living with MLTCs before their last year of life (see Supplementary Table S2). The most common were hypertension (21.2%), cancer (18.7%), and arrhythmias (14.1%) (Table 2). Compared with those without MLTCs (Table 1), individuals with MLTCs were older (59.1% with MLTCs were aged ≥80 years versus 47.9% without), more likely to be female (51.9% with MLTCs were female versus 49.7% without), and more socioeconomically deprived (25.3% with MLTCs in the most deprived quintile versus 23.7% without). The groups were evenly distributed with regard to rurality. There were differences in recording of ethnicity, which was missing for 6.6% with MLTCs and 20.9% without. Those with MLTCs were less likely to die at home (25.0% with MLTCs versus 33.2% without).

**Table 2.** Prevalence of conditions identified in decedents before the last year of life

Long-term condition	Decedents with condition, *n*	Prevalence in decedent cohort, %
Hypertension	63 325	21.2
Cancer	56 060	18.7
Arrhythmia	42 305	14.1
Coronary artery disease	41 480	13.9
Diabetes	36 885	12.3
Chronic obstructive pulmonary disease	34 986	11.7
Dementia	29 902	10.0
Drug or alcohol misuse	24 168	8.1
Stroke or TIA	23 373	7.8
Heart failure	21 162	7.1
Osteoarthritis	16 018	5.4
Heart valve disorders	14 117	4.7
Anaemia	13 631	4.6
Asthma	11 376	3.8
Osteoporosis	9957	3.3
Peripheral artery disease	9947	3.3
Thyroid disorders	9480	3.2
Venous thromboembolic disease	6988	2.3
Chronic liver disease	6936	2.3
Connective tissue disease	6932	2.3
Depression	5731	1.9
Anxiety	5508	1.8
Epilepsy	5113	1.7
Peptic ulcer	5093	1.7
Chronic kidney disease	5050	1.7
Parkinson’s disease	4424	1.5
Aneurysm	3309	1.1
Bronchiectasis	3212	1.1
Gout	3060	1.0
Paralysis	2834	0.9
Schizophrenia	2615	0.9
Inflammatory bowel disease	1989	0.7
Vision impairment that cannot be corrected	1419	0.5
Multiple sclerosis	1315	0.4
Hearing impairment that cannot be corrected	1225	0.4
Bipolar disorder	1175	0.4
Chronic pancreatic disease	986	0.3
Congenital and chromosomal abnormalities	935	0.3
Chronic primary pain	622	0.2
Peripheral neuropathy	615	0.2
Addison’s disease	393	0.1
Post-traumatic stress disorder	208	0.1
HIV or AIDS	196	0.1
Ménière’s disease	175	0.1
Tuberculosis	164	0.1
Endometriosis	131	0.04
Eating disorder	108	0.04
Autism	99	0.03
Cystic fibrosis	83	0.03

AIDS = acquired immunodeficiency syndrome. HIV = human immunodeficiency virus. TIA = transient ischaemic attack.

### Use of unscheduled care in the last year of life

The 299 361 decedents had a total of 3 256 021 contacts with unscheduled care services in their last year of life over the 5-year study period. Highest usage was recorded for hospital admissions (774.0 per 1000 decedents), then SAS contacts (770.4 per 1000 decedents), A&E attendances (678.2 per 1000 decedents), GPOOH contacts (569.0 per 1000 decedents), then NHS 24 contacts (558.4 per 1000 decedents) (Table 3).

**Table 3.** Number of decedents who contacted each unscheduled care service, median number of contacts, and number of contacts per 1000 deaths for those with and without MLTCs

Category	Decedents who had contact with each service in the last year of life, *n* (% of decedents in each group)	Median number of contacts in the last year of life (IQR)	Contacts per 1000 deaths, *n*
**Any unscheduled care service**			
<2 long-term conditions	155 610 (95.6)	7 (4–13)	956.0
≥2 long-term conditions	132 685 (97.1)	10 (5–18)	971.4
All decedents	288 295 (96.3)	8 (4–15)	963.0
**Admissions**			
<2 long-term conditions	120 875 (74.3)	3 (2–6)	742.6
≥2 long-term conditions	110 831 (81.1)	4 (2–7)	811.4
All decedents	231 706 (77.4)	4 (2–7)	774.0
**Scottish Ambulance Service**			
<2 long-term conditions	120 932 (74.3)	1 (1–2)	743.0
≥2 long-term conditions	109 697 (80.3)	2 (1–3)	803.1
All decedents	230 629 (77.0)	2 (1–3)	770.4
**Accident and emergency**			
<2 long-term conditions	104 863 (64.4)	1 (1–2)	644.3
≥2 long-term conditions	98 153 (71.9)	2 (1–3)	718.6
All decedents	203 016 (67.8)	2 (1–3)	678.2
**GP out of hours**			
<2 long-term conditions	83 422 (51.3)	2 (1–4)	512.5
≥2 long-term conditions	86 910 (63.6)	3 (1–5)	636.3
All decedents	170 332 (56.9)	2 (1–4)	569.0
**NHS 24**			
<2 long-term conditions	81 567 (50.1)	2 (1–3)	501.1
≥2 long-term conditions	85 605 (62.7)	2 (1–3)	626.7
All decedents	167 172 (55.8)	2 (1–3)	558.4

MLTCs = multiple long-term conditions.

**Figure 1.**
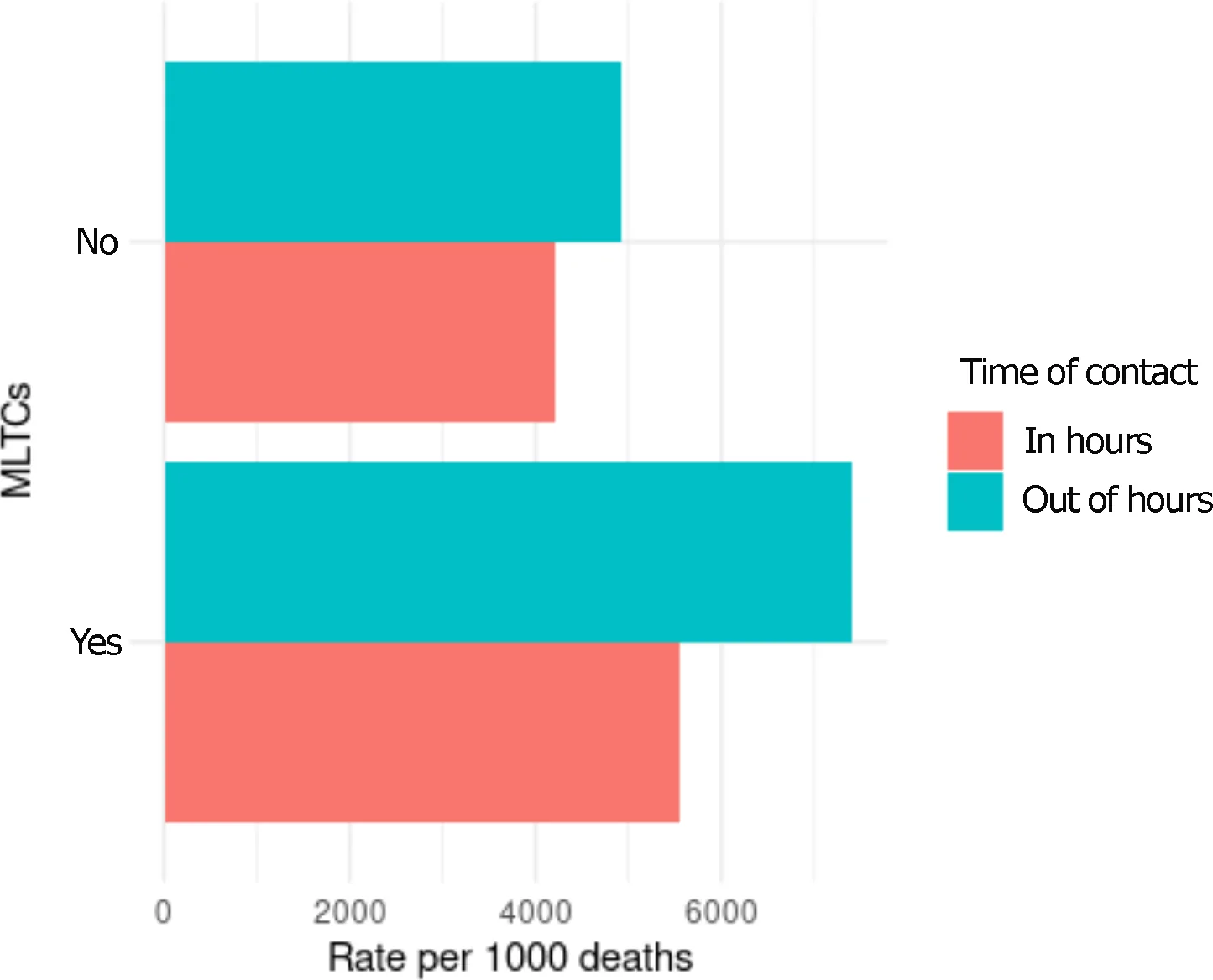
Contacts with unscheduled care services in and out of hours for those with and without MLTCs. MLTCs = multiple long-term conditions.

A higher proportion of people with MLTCs used unscheduled care in the last year of life (97.1%), compared with people without MLTCs (95.6%, Table 3). The number of contacts with unscheduled care in the last year of life was higher for individuals with MLTCs (971.4 per 1000 decedents with MLTCs versus 956.0 per 1000 decedents without). Use of unscheduled care services out of hours was higher for those with MLTCs (Figure 1: 7419.3 per 1000 decedents with MLTCs versus 4914.1 per 1000 decedents without).

**Figure 2.**
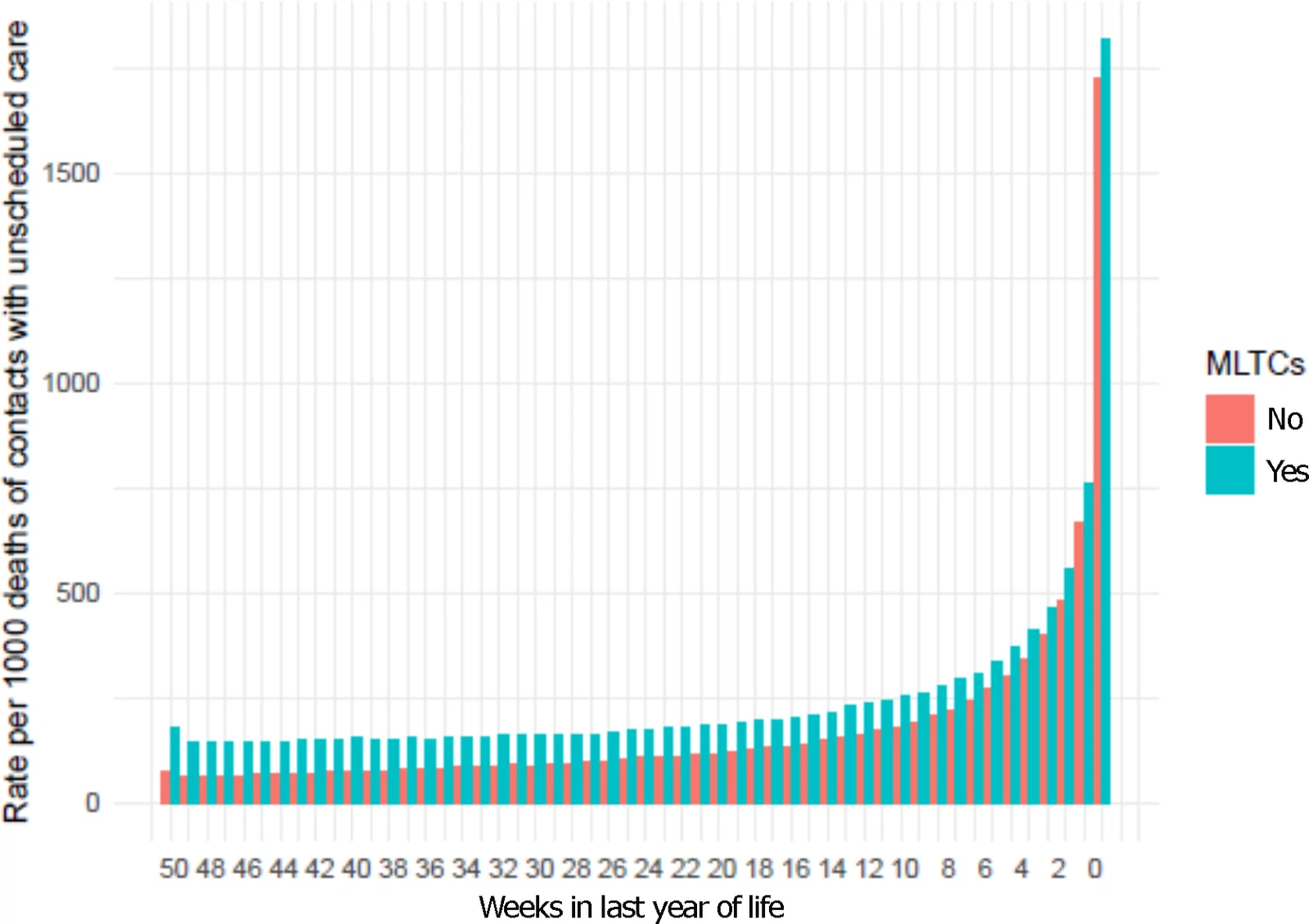
Unscheduled care contacts in the last year of life, stratified for MLTCs. MLTCs = multiple long-term conditions

Unscheduled care usage increased progressively with proximity to death for all decedents (Figure 2). Those with MLTCs typically used unscheduled care earlier in their last year of life (median 13 weeks before death, IQR 3–30 versus median 8 weeks before death, IQR 3–30 for those without MLTCs). Both groups had a surge in unscheduled care usage in the final few weeks of life, with the biggest rise in the final week. When stratified for type of unscheduled care, those with MLTCs consistently had higher rates of GPOOH, NHS 24, and acute admissions at all time points (see Supplementary Figures S1A–S1C). However, for SAS and A&E, decedents without MLTCs used these services more in the final week of life (see Supplementary Figures S1D and S1E).

### Logistic regression modelling

Ethnicity could not be included as a variable within regression modelling owing to a high proportion of missing data (*n* = 43 105 [14.4%] decedents). There were 391 decedents (0.1%) with missing SIMD 2020 and 8-fold Urban Rural classification who were therefore also not included in regression modelling.

### Factors influencing use of unscheduled care: demographics

Males were more likely to use unscheduled care services than females (aOR 1.16, 95% CI = 1.11 to 1.20). Older age was associated with increased unscheduled care usage (aged 70–79 years aOR 5.07, 95% CI = 4.36 to 5.86), but this difference was less marked in the oldest ages (aged ≥80 years aOR 3.20, 95% CI = 2.77 to 3.68). Unscheduled care usage was correlated with socioeconomic status, with higher rates seen in people who were more socioeconomically deprived (most deprived quintile aOR 1.27, 95% CI = 1.20 to 1.35). Overall, people living in the most rural areas used less unscheduled care than their urban counterparts (most rural level aOR 0.53, 95% CI = 0.49 to 0.59) (see Supplementary Table S3).

### Factors influencing use of unscheduled care: MLTCs

When adjusted for available sociodemographic factors, presence of MLTCs was associated with unscheduled care use in the last year of life (aOR 1.51, 95% CI = 1.45 to 1.57) (Table 4). MLTCs consistently accounted for higher use of each type of unscheduled care for decedents in the last year of life, with aORs of a similar magnitude demonstrated (see Supplementary Table S4).

**Table 4.** Logistic regression for the influence of the presence of multiple long-term conditions on use of unscheduled care, adjusted for sociodemographic factors: age, sex, SIMD 2020 quintile, and Urban Rural Classification 2020

Category	Total decedents, *n*	Used any unscheduled care in last year of life, *n* (%)	Unscheduled care use, unadjusted OR (95% CI)	Unscheduled care use, adjusted OR (95% CI)
**Multiple long-term conditions**				
<2	162 768	155 610 (95.6)	**—**	—
≥2	136 593	132 685 (97.1)	1.56 (1.50 to 1.63)	1.51 (1.45 to 1.57)
**Age, years**				
18–29	2085	1858 (89.1)	—	—
30–39	4307	3963 (92.0)	1.41 (1.18 to 1.68)	1.37 (1.14 to 1.63)
40–49	8925	8402 (94.1)	1.96 (1.66 to 2.31)	1.87 (1.58 to 2.21)
50–59	19 418	18 583 (95.7)	2.72 (2.33 to 3.17)	2.64 (2.25 to 3.07)
60–69	36 768	35 648 (97.0)	3.89 (3.34 to 4.51)	3.71 (3.18 to 4.31)
70–79	69 270	67 562 (97.5)	4.83 (4.17 to 5.58)	4.53 (3.90 to 5.24)
≥80	158 588	152 279 (96.0)	2.95 (2.56 to 3.38)	2.79 (2.41 to 3.21)
**Sex**				
Female	151 751	145 783 (96.1)	—	—
Male	147 610	142 512 (96.5)	1.14 (1.10 to 1.19)	1.16 (1.11 to 1.21)
**SIMD 2020 quintile^a^**				
5 (least deprived)	44 639	42 758 (95.8)	—	—
4	52 077	49 966 (95.9)	1.04 (0.98 to 1.11)	1.05 (0.98 to 1.12)
3	61 948	59 589 (96.2)	1.11 (1.05 to 1.19)	1.15 (1.08 to 1.23)
2	67 253	64 964 (96.6)	1.27 (1.19 to 1.35)	1.23 (1.16 to 1.32)
1 (most deprived)	73 053	70 492 (96.5)	1.22 (1.14 to 1.29)	1.23 (1.16 to 1.31)
**Urban Rural Classification 2020^b^**				
1 (most urban)	105 442	101 521 (96.3)	—	—
2	106 735	103 406 (96.9)	1.28 (1.22 to 1.33)	1.25 (1.20 to 1.31)
3	26 132	25 367 (97.1)	1.35 (1.25 to 1.45)	1.35 (1.25 to 1.46)
4	5799	5621 (96.9)	1.27 (1.10 to 1.48)	1.27 (1.09 to 1.48)
5	4598	4403 (95.8)	0.90 (0.78 to 1.04)	0.88 (0.76 to 1.02)
6	32 213	31 093 (96.5)	1.15 (1.08 to 1.23)	1.16 (1.08 to 1.24)
7	8684	8352 (96.2)	1.04 (0.93 to 1.17)	1.05 (0.94 to 1.18)
8 (most rural)	8614	8006 (92.9)	0.55 (0.51 to 0.60)	0.54 (0.49 to 0.60)

^a^ *n *= 391 decedents did not have SIMD 2020 or Urban Rural Classification data available and therefore were excluded from this logistic regression. ^b^Urban Rural Classification categories: 1 = ≥125 000 people; 2 = 10 000–124 999 people; 3 = 3000–9999, within a 30-minute drive to a settlement of ≥10 000; 4 = 3000–9999, with a 30–60-minute drive to a settlement of ≥10 000; 5 = 3000–9999, with >60-minute drive to a settlement of ≥10 000; 6 = <3000 people, within a 30-minute drive to a settlement of ≥10 000; 7 = <3000 people, with a 30–60-minute drive to a settlement of ≥10 000; and 8 = <3000 people, with >60-minute drive to a settlement of ≥10 000. SIMD = Scottish Index of Multiple Deprivation.

## Discussion

### Summary

This retrospective cohort study enabled detailed characterisation of Scotland’s population who were living with MLTCs before their last year of life, and their usage of unscheduled health care. The study has highlighted various demographic and clinical factors associated with unscheduled care usage, including the extent, timing, and types of services accessed. Key findings are that decedents with pre-existing MLTCs had higher rates of unscheduled care service usage across all service types, and that contacts started sooner in the last year of life and were more likely to be in the out-of-hours period compared with people without MLTCs.

### Strengths and limitations

This study utilised robust methodology to link and analyse several routinely collected healthcare datasets. It is the first, to our knowledge, to quantify the whole population of Scotland living with MLTCs before death and describe their unscheduled care usage. Existing definitions of MLTCs within research literature are wildly heterogeneous, and the Delphi study from which we based our definition of MLTCs sought to address this.^18^ Despite using the Delphi-derived consensus list, adaptations were required owing to data availability (see Supplementary Table S1), which should be of use to future researchers. Describing the population ‘living with’ MLTCs, rather than ‘dying of’, circumvents known under-reporting of MLTCs on death certificates, and better represents lived experience, further enhanced through the strong integration of the PAG.^21^

This study focused on unscheduled care service usage only, as wider datasets of interest were unavailable, including general practice ‘in hours’, community nursing, social care, and specialist palliative care. Primary care settings provide the mainstay of community palliative care, which is an important omission to acknowledge.^22^ Furthermore, primary care data would have enabled enhanced clinical characterisation of decedents, as most long-term conditions are managed in the community,^23^ and enabled more complete identification of decedents’ ethnicity, which influences healthcare usage at the end of life.^24,25^ Improving access to integrated datasets, particularly primary care, should be a priority for research policymakers and data providers. Additional detail around the reasons for and outcomes of contacts with unscheduled care services would have been desirable, but was not feasible owing to missing data. Thus, no inferences could be drawn about the nature or value of contacts.

### Comparison with existing literature

Previous research has examined unscheduled care usage for individuals living with particular conditions such as cancer.^6,7^ However, this ‘single condition’-focused approach does not reflect the reality for the many patients who live with multiple competing and interacting conditions. There is growing recognition that to be able to meet the needs of our ageing and increasingly multimorbid population, a ‘whole-person’ (and, indeed, ‘all-conditions’) approach is needed, rather than services designed to deliver single-condition, guideline-based care.^26^ Figure 2 and Supplementary Figures S1A–S1E show differing patterns of unscheduled care use for individuals with MLTCs compared with those without. Those with MLTCs had persistently higher use of unscheduled care throughout the final year, suggesting a longer period with urgent unmet needs. However, those without MLTCs had subtly higher use of SAS and A&E in the final week, which perhaps reflects acute illnesses contributing to death in those without pre-diagnosed long-term conditions.

This study confirms that most unscheduled care contacts are out of hours.^7^ The extent to which decedents’ needs could have been met by usual care providers rather than unfamiliar unscheduled care teams remains unknown. However, it is well recognised that poor care continuity and fragmented service delivery near the end of life threaten quality of care delivered, and that poorly controlled symptoms are frequently responsible for unscheduled care contacts.^8^ Potential solutions, such as single points of access and care coordination for those nearing the end of life, have been established to ensure that people receive an urgent care response that is targeted to their particular needs; although there is geographical variance in their availability.^8^

This study also highlights that while MLTCs influence unscheduled care usage, there is a wider complex interplay with sociodemographic profiles. People living with high socioeconomic deprivation develop MLTCs earlier^27^ and typically die earlier^28^ than their counterparts. Furthermore, people living with socioeconomic deprivation use health care differently towards the end of life and, most importantly, their needs are often not met.^29,30^ This study’s findings that people with MLTCs in areas of high socioeconomic deprivation are high users of unscheduled care near the end of life raises the question of whether they are accessing the planned care they need. Further research is needed, integrating lived experiences alongside findings from routine data, to better design services that meet everyone’s needs and reduce health inequalities.^31,32^

### Implications for research and practice

This study has illuminated the scale of the population with MLTCs who are nearing the end of life in Scotland, and highlighted that they are intensive and regular users of unscheduled care services. People with MLTCs are predicted to become the highest users of general and specialist palliative and end-of-life care services.^14^ Integration of a palliative care approach into the care of people with a range of serious health conditions could helpfully reduce the unscheduled care usage and align care better with person-centred preferences, a Realistic Medicine approach.^33,34^ Given the immense and growing pressures on unscheduled care services, and the profile this has with policymakers,^35^ this present study provides additional evidence that care for people with MLTCs at the end of life deserves serious attention. Unprecedented financial pressures mean that redesigning existing services to meet urgent unmet care needs, within both in-hours and out-of-hours periods, may be more realistic than new investment. Given that the majority of people spend the majority of their last year of life in community settings, and that they typically wish to remain there, any such development should have primary care at its heart.

## Data availability

The datasets used in this study are not publicly available, though can be requested through further arrangements with eDRIS - phs.eDRIS@phs.scot.

## Data

The data are not publicly available, though can be requested via email through further arrangements with eDRIS: phs.eDRIS@phs.scot.
